# Evaluation of an Agricultural Meteorological Disaster Based on Multiple Criterion Decision Making and Evolutionary Algorithm

**DOI:** 10.3390/ijerph15040612

**Published:** 2018-03-28

**Authors:** Xiaobing Yu, Xianrui Yu, Yiqun Lu

**Affiliations:** 1 Collaborative Innovation Center on Forecast and Evaluation of Meteorological Disasters, Nanjing University of Information Science & Technology, Nanjing 210044, China; 2 School of Management and Engineering, Nanjing University of Information Science & Technology, Nanjing 210044, China; 20171213556@nuist.edu.cn (X.Y.); luyiqun@nuist.edu.cn (Y.L.)

**Keywords:** disaster evaluation, evaluation model, TOPSIS, Analytical Hierarchy Process (AHP), differential evolution

## Abstract

The evaluation of a meteorological disaster can be regarded as a multiple-criteria decision making problem because it involves many indexes. Firstly, a comprehensive indexing system for an agricultural meteorological disaster is proposed, which includes the disaster rate, the inundated rate, and the complete loss rate. Following this, the relative weights of the three criteria are acquired using a novel proposed evolutionary algorithm. The proposed algorithm consists of a differential evolution algorithm and an evolution strategy. Finally, a novel evaluation model, based on the proposed algorithm and the Technique for Order of Preference by Similarity to Ideal Solution (TOPSIS), is presented to estimate the agricultural meteorological disaster of 2008 in China. The geographic information system (GIS) technique is employed to depict the disaster. The experimental results demonstrated that the agricultural meteorological disaster of 2008 was very serious, especially in Hunan and Hubei provinces. Some useful suggestions are provided to relieve agriculture meteorological disasters.

## 1. Introduction

China is a country with a large population and rapid economic development. Agriculture is, not only related to the daily lives of local residents, but also plays a significant role in the sustainable development of the country and the stability of the global grain market. A meteorological disaster is one of the most serious types of natural disasters. It can have devastating effects on water supply, crop production, and the rearing of livestock. It may lead to famine, malnutrition, epidemics, and the displacement of large populations from one area to another [1,2]. More importantly, its impact on agriculture is enormous. Such disasters also cause significant harm to economies, societies, and environments. They have a large influence on the development of China, and have become bottlenecks for the sustainable development of the national economy [3,4,5]. Therefore, it is useful to learn about the realities of meteorological disasters. It can help us to take more useful measures in order to protect agriculture from being destroyed by these disasters.

There are currently two main approaches that are used to evaluate meteorological disasters: The fuzzy comprehensive evaluation method and the risk probabilistic method [6,7,8,9,10]. The probabilistic method is widely used in the financial and insurance sectors to assess potential losses. The fuzzy comprehensive evaluation method constructs an index system, which employs fuzzy mathematics and the analytic hierarchy process (AHP), according to the formation mechanism of the meteorological disaster. This method has been widely used to evaluate agricultural disasters. However, the weights of the indexes are generally acquired using the AHP theory. Since the evaluation of a meteorological disaster involves the use of many indexes, it can be regarded as a multiple criterion decision making problem. This technique can be employed to solve this problem. In order to make a fair evaluation, a model that uses multiple criterion decision making methods is proposed. The technique mainly includes a novel evolutionary algorithm and the Technique for Order of Preference by Similarity to Ideal Solution (TOPSIS). Firstly, the meteorological disaster indexes are established. In order to solve for the weights, the weight acquisition is converted into a constraint optimization problem. This is a novel idea that is used to solve for the weights. In this article, a novel algorithm—based on differential evolution (DE)—and evolution strategy are combined to design an optimization algorithm. Following this, the algorithm is used to acquire the weights of the indexes. Lastly, a novel evaluation model—based on the proposed algorithm and the TOPSIS—is put forward to estimate the meteorological disaster in China. The geographical information system (GIS) technique is used to depict the disaster. 

A disaster that caused unprecedented low temperatures, rain, snow, and ice occurred in Southern China from 10 January 2008 to 5 February 2008. As a result of its unusual persistence and intensity, this disaster caused great losses for the national economy, especially in transportation, energy supply, electric power transmission, communication facilities, agricultural and ecological systems, and peoples’ lives across most of the country. In order to make a comprehensive evaluation, the disaster that occurred during this year was selected. The experimental results have demonstrated that the meteorological disaster of 2008 was very serious. In order to relieve agricultural meteorological disasters, some useful suggestions are provided.

The paper is organized as follows: The related works are presented in Section 2. The AHP, the TOPSIS, and DE are briefly introduced in Section 3, and the proposed algorithm and evaluation model are put forward. The experiments are conducted, based on the standard benchmarks and the disaster data, in Section 4. A discussion is given in Section 5. The conclusions are made in Section 6. 

## 2. Related Works

The effects of agricultural disasters have been studied in recent decades. The strengthening of disaster risk assessment is necessary. This is important in order to reduce the influence of and losses from meteorological disasters. There are currently two principal methods that are used to evaluate meteorological disasters: the fuzzy comprehensive evaluation method [6,7] and the risk probabilistic method.

The meteorological disaster risk of Southern China was analyzed from 1949–2012 [8]. Drought and waterlogging disasters were analyzed in Anhui Province using the fuzzy comprehensive evaluation method [9]. Rainstorm and flood disaster losses were evaluated in the Chinese mainland from 2004–2009 [10]. Since urban areas are susceptible to natural disasters [11], the AHP and the GIS are combined to map landslide, flood, and seismic hazards [12,13]. The flood hazard assessment model—based on the AHP method—is proposed for urban areas [14]. The grey incidence multiple-attribute decision model is used to evaluate China’s regional rainstorm and flood disasters [15]. Based on a series of drought data that was acquired from 1952–2011, the evolutionary characteristics and the changing trends of agricultural drought disasters are analyzed using quantitative and qualitative methods [16].

Based on probability distribution functions, a methodology for risk analysis, assessment, and the combination of drought disasters under the different irrigational levels in Baicheng City is presented [17]. The risk of China’s agriculture drought disaster is evaluated using the higher spatial resolution of the county unit, based on the information diffusion theory [18]. An agricultural drought intensity index, based on rainfall and the demand for water for crops, is proposed [19]. The quantitative relationship between the hazard-induced factors of extreme meteorological disasters and the affected area in the tail of the distribution is depicted [20]. Based on the least squares method, the area that was affected by meteorological disasters, especially by floods and droughts, increased significantly in China during 1950–2013 [21]. The different kinds of meteorological disasters, including floods, droughts, tropical storms, hail disasters, and snowstorms, are analyzed using the grey cluster model [22]. The features of the major natural disasters that occurred in the Chinese mainland between 1980–2011 were explored using a regression analysis [23]. A crop yield-climate analysis and a regression analysis are employed to analyze and quantify the relationship between the fluctuation of maize yield and agro-meteorological disasters [24].

## 3. Methodology

### 3.1. AHP

AHP is used to apply multiple criterion decision making to real applications [25,26]. In the AHP, multiple pairwise comparisons come from a standardized comparison scale consisting of nine levels. Suppose that *C* = {*C_k_|k* = 1, 2, …, *n*} is the set of criteria. An evaluation matrix can be obtained in which every element $a_{ij}\left(i,j=1,\;2,\ldots ,\;n\right)$ represents the relative weights of the criteria *C*. If matrix *A* is completely consistent, then it has complied with following condition:

(1) $$a_{ii}=\frac{w_{i}}{w_{i}}=1$$

(2) $$a_{ji}=\frac{w_{j}}{w_{i}}=\frac{1}{a_{ij}}$$

(3) $$a_{ij}a_{jk}=\frac{w_{i}}{w_{j}}\times \frac{w_{j}}{w_{k}}=\frac{w_{i}}{w_{k}}=a_{ik}$$

According to the above properties, the following equations can be obtained:

(4) $$\sum_{k=1}^{n}\left(a_{ik}w_{k}\right)=\sum_{k=1}^{n}\left(\frac{w_{i}}{w_{k}}\right)w_{k}=nw_{i},i=1,\;2,\ldots ,\;n$$

(5) $$\sum_{i=1}^{n}\left|\sum_{k=1}^{n}(a_{ik}w_{k})-nw_{i}\right|=0$$

In other words, if a judgment matrix meets Equation (5), then it is completely consistent. However, it is very difficult to achieve this condition in real applications. In fact, the matrix must just meet the satisfactory consistency, then Equation (4) can be converted into the following format:

(6) $$\min CIF(w)=\frac{\sum_{i=1}^{n}\left|\sum_{k=1}^{n}(a_{ik}w_{k})-nw_{i}\right|}{n}$$

(7) $$0<w_{k}<1,\;\sum_{k=1}^{n}w_{k}=1$$

A smaller consistent inspection function (*CIF*) indicates a more consistent matrix *A*. Therefore, the weight acquisition is converted into the single objective optimization with a constraint. The objective is to minimize the *CIF*, and the constraint is $0<w_{k}<1$, $\sum_{k=1}^{n}w_{k}=1$.

To solve the constraint optimization problem, the equality constraints are generally converted to inequality constraints as follows:

(8) $$\left|\sum_{k=1}^{n}w_{k}-1\right|-\delta \leq 0$$

where δ is the tolerance value for the equality constraints. Generally speaking, δ is set to 0.0001. The absolute value operator can then be removed by transforming Equation (8) into the inequality constraints, in which *k* ranges from one to the number of criteria.

(9) $$-\delta \leq \sum_{k=1}^{n}w_{k}-1\leq \delta \to \left\{ \begin{array}{l} \sum_{k=1}^{n}w_{k}-1\leq \delta \\ -(\sum_{k=1}^{n}w_{k}-1)\leq \delta \end{array}\right.$$

The constraint violation (*CV*) can be briefly expressed as follows:

(10) $$CV(\vec{x})=\max((\sum_{k=1}^{n}w_{k})-1-\delta ,0)+\max(-(\sum_{k=1}^{n}w_{k})-1)-\delta ,0)$$

### 3.2. Proposed Algorithm Based on DE and Evolution Strategy

#### 3.2.1. Conventional DE

DE is a population-based algorithm [27]. There are three operators in DE: The mutation, the crossover, and the selection.

(1) Mutation

DE employs the mutation operation to generate a trial vector *V_i_*. *V_i_* can be produced by mutation strategies. The following mutation strategy was proposed first, and is one of the most successful strategies [28,29]. Thus, it is adopted.

DE/rand/1:

(11) $$V_{i}=X_{r_{1}^{i}}+F.(X_{r_{2}^{i}}-X_{r_{3}^{i}})$$

The indexes $r_{1}^{i}$, $r_{2}^{i}$ are randomly generated within the range [0,*NP*], where *NP* is the population size. $X_{r_{1}^{i}}$, $X_{r_{2}^{i}}$, and $X_{r_{3}^{i}}$ are the current vectors. The mutation scale factor *F* is used to control the amplification of the differential variations. *V_i_* is the mutation vector.

(2) Crossover

The crossover operation is employed on $V_{i}=\left\{v_{i}^{1},v_{i}^{2},\ldots ,v_{i}^{D}\right\}$ and $X_{i}=\left\{x_{i}^{1},x_{i}^{2},\ldots ,x_{i}^{D}\right\}$ to produce a trial vector $U_{i}=\left\{u_{i}^{1},u_{i}^{2},\ldots ,u_{i}^{D}\right\}$ as follows:

(12) $$u_{i}^{j}=\{\begin{array}{c} v_{i}^{j}\;if\;rand_{j}\left[0,1\right)\leq CR\;or\;\left(j=j_{rand}\right)\;\\ x_{i}^{j}\;others\; \end{array}$$

*CR* ∈ [0,1] is the crossover rate, which has to be determined by the user. The index *j_rand_* ∈ [1,*D*] ensures that the trial vector *U_i_* will be different from *x_i_*.

(3) Selection

The trial vector *U_i_* is compared with *x_i_*. If the fitness value of *u_i_* is better than that of *x_i_*, then *u_i_* will replace *x_i_*. Otherwise, the old value *x_i_* is retained.

(13) $$x_{i}=\{\begin{array}{c} u_{i}\;if\;f\left(u_{i}\right)\leq f\left(x_{i}\right)\;\\ x_{i}\;otherwise\; \end{array}$$

#### 3.2.2. The Proposed Algorithm Based on DE

If a solution meets the requirements of the constraints in Equation (8), it is a feasible solution. If it does not meet the requirements of the constraints in Equation (8), the solution is an infeasible solution. In conventional DE, greedy selection is used. If the trial vector *U_i_* yields a better function value than *x_i_*, then *u_i_* will replace *x_i_* and enter the population of the next generation. If the trial vector *U_i_* does not yield a better function value than *x_i_*, the old value *x_i_* is retained. However, this operation cannot ensure that all of the feasible solutions will have better chances of survival than the infeasible solutions. In order to improve more of the feasible solutions’ chances of survival, a selection scheme that is similar to an evolution strategy (ES) is utilized, since ES have strong theoretical support [30]. Firstly, the offspring are generated by DE. Following this, the parent population and the offspring population are combined to form a mating pool. The mating pool is then divided into feasible solutions and infeasible solutions. The feasible and infeasible solutions are then sorted into ascending order, according to the fitness value and the *CV*, respectively. If the number of feasible solutions is greater than the population size, the population that will enter the next generation will be selected directly from these solutions. If the number of feasible solutions is smaller than the population size, some of the infeasible solutions will be chosen to enter the next generation.

According to the above discussion, the main procedure of the proposed algorithm is presented as follows: 

Step 1: Initialize the parameters. *Max*_*FES*: maximum number of function evaluations, *NP*: population size, mutation scale factor *F*, and crossover constant *CR*.

Step 2: Set *G* = 1 and randomly generate *NP* individuals from *pop* = {*X*_1,*G*_, *X*_2,*G*_,…,*X*_*NP*,*G*_} with $X_{i,G}=\{X_{i,G}^{1},\ldots ,X_{i,G}^{D}\},\;i=1,2,\ldots ,NP$ uniformly distributed in the range $\left[X_{\min},X_{\max}\right]$. *D* is the dimension of *X*.

Step 3: Calculate the fitness value and the *CV*.

Step 4: *FES = FES + NP*

Step 5: If the stopping criterion is not met

Step 5.1: Generate vector $V_{i}$ according to the population pop using Equation (11)

Step 5.2: Generate vector $U_{i}$ using Equation (12).

Step 5.3: If the trial vector $U_{i}$ is outside the boundary, then randomly generate them within the search space

Step 5.4: Calculate the fitness value and *CV* of $U_{i}$

Step 5.5: p = (*pop*, $U_{i}$);

  (pf, pinf) = divide (p);// pf: feasible solutions and pinf: infeasible solutions

  Sort pf by the fitness value

  Sort pinf by the CV

  If(size(pf) ≥ *NP*)

  pop = pf(1:*NP*);

  Else

  pop = pf + pinf(*NP*−size(pf));

  End

Step 5.6: *FES = FES + NP*; 

Step 6: End while

### 3.3. Proposed Evaluation Model Based on TOPSIS

#### 3.3.1. TOPSIS

TOPSIS is one of the multiple criterion decision making methods that are used to evaluate alternatives [31,32,33]. It consists of the following steps: 

Step 1: Obtain the decision matrix. 

The number of alternatives is *m* and the number of criteria is *n*. The decision matrix *f_ij_* (*i* = 1, 2, …, *n*; *j* = 1, 2, …, *m*), with *n* rows and *m* columns, will be obtained. *f_ij_* is a value that indicates the performance rating of each *j*th alternative with respect to each *i*th criterion.

Step 2: Normalize the decision matrix.

According to Equation (14), the normalized value *f_ij_* is calculated as follows:

(14) $$r_{ij}=\frac{f_{ij}}{\sqrt{\sum_{j=1}^{m}f_{ij}^{2}}},i=1,2,\ldots ,n;j=1,2,\ldots ,m$$

Step 3: Calculate the weighted normalized decision matrix.

The matrix is calculated by multiplying normalized decision matrix. Its weights are presented as follows:

(15) $$v_{ij}=w_{i}\times r_{ij}$$

where $w_{i}$ is the weight of the *i*th criterion and $\sum_{i=1}^{n}w_{i}=1$.

Step 4: Find the negative-ideal and positive-ideal solutions.

(16) $$A^{-}=\left\{v_{1}^{-},v_{2}^{-},\ldots ,v_{n}^{-}\right\}=\left\{\left(\min v_{ij}|i\in I^{\prime }\right),\left(\min v_{ij}|i\in I^{\prime \prime }\right)\right\}$$

(17) $$A^{+}=\{\upsilon_{1}^{+},\upsilon_{2}^{+},\ldots ,\upsilon_{n}^{+}\}=\{(\max\upsilon_{ij}|i\in I^{\prime }),(\min\upsilon_{ij}|i\in I^{\prime })\}$$

where *I*’ is associated with the cost criteria and *I*’’ is associated with the benefit criteria.

Step 5: Calculate the *n*-dimensional Euclidean distance.

The separation of each algorithm from the ideal solution is presented as follows:

(18) $$D_{j}^{+}=\sum_{i=1}^{n}d\left(v_{ij},v_{j}^{+}\right)$$

The separation of each algorithm from the negative solution is presented as follows:

(19) $$D_{j}^{-}=\sum_{i=1}^{n}d\left(v_{ij},v_{j}^{-}\right)$$

Step 6: Calculate the relative closeness to the ideal solution.

The relative closeness of the *j*th alternative is defined as follows:

(20) $$CC_{j}=\frac{D_{j}^{-}}{D_{j}^{-}+D_{j}^{+}},i=1,2,\ldots ,m$$

Step 7: Rank the alternative order.

The *CC_j_* is between zero and one. A larger *CC* indicates a better alternative *j*.

#### 3.3.2. The Proposed Model

The proposed model for evaluating agricultural meteorological disasters (composed of the AHP, the evolutionary algorithm, and the TOPSIS) has the following three phases:

(1) Identify the criteria and acquire the data

In the first phase, the provinces and the criteria that will be used in the ranking are determined and the decisional hierarchy is formed. The AHP model is established. The objective is in the first level, the criteria are contained in the second level, and the provinces are contained in the third level. According to the statistical data from the Chinese Ministry of Agriculture, the criteria of the multi-objective decision include the disaster rate (*C*1), the inundated rate (*C*2), and the complete loss rate (*C*3). Out of the three criteria, the complete loss rate, meaning that no gains have been realized as a result of the disasters, is regarded as the most serious. The inundated rate, which can cause some loss, is regarded as being more serious. The disaster rate is regarded as being serious. The larger the planting area is, the larger the loss is. It is unfair to directly use the areas as criteria. Thus, the rate is adopted in order to obtain fair results.

(21) $$C1=\frac{disaster\;area}{planting\;area}\times 100\%$$

(22) $$C2=\frac{inundated\;area}{planting\;area}\times 100\%$$

(23) $$C3=\frac{complete\;loss\;area}{planting\;area}\times 100\%$$

(2) Calculate the criteria weights using the proposed algorithm

In this phase, the pairwise comparison matrix is constructed in order to acquire the criteria weights. The experts make their evaluations using the scale (1~9). The evaluation matrix can be obtained in order to determine the weights of the criteria. According to Equations (6) and (7), the calculation of the weights can be converted to a single constraint optimization problem. The proposed algorithm can be used to solve the optimization problem.

(3) Evaluate the disaster and determinate the final ranks using TOPSIS

**Figure 1:**
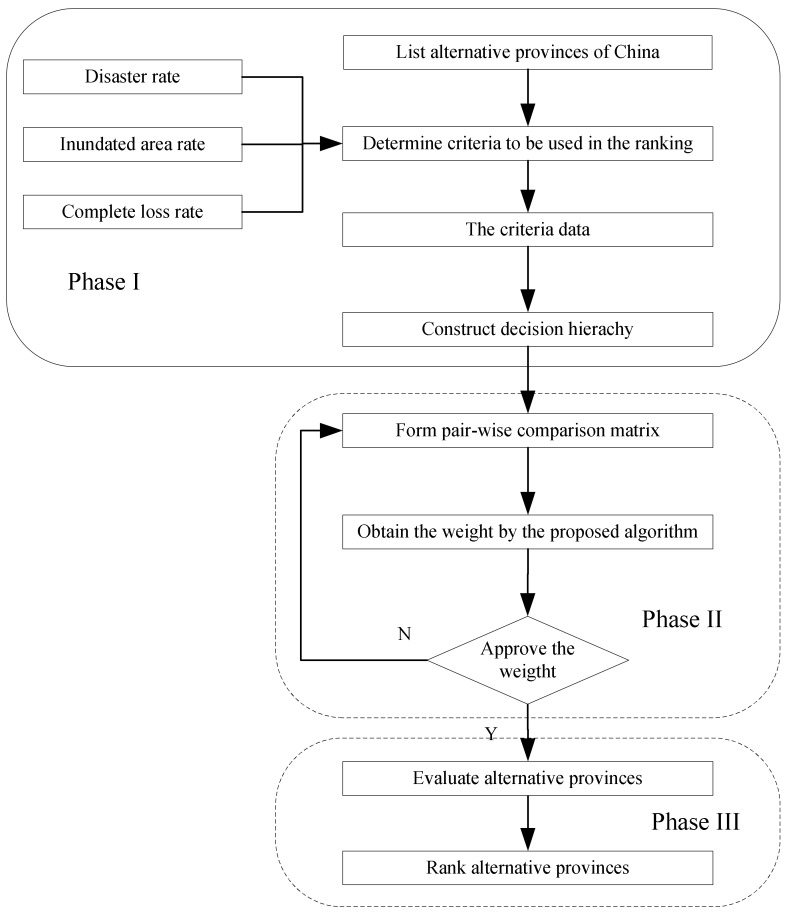
The proposed model.

There are 31 provinces and cities and the number of criteria is three. Therefore, the decision matrix *f_ij_* (*i* = 1, 2, …, 3; *j* = 1, 2, …, 31) can be obtained. Following this, the disaster evaluation is determined by using the TOPSIS in the third phase, according to Equations (14)–(20). The province rankings are determined, according to the *CC* that is calculated using TOPSIS, in descending order. Figure 1 presents the whole process.

## 4. Results

### 4.1. Algorithm Experiment

In order to verify the performance of the proposed algorithm, eleven benchmark functions are selected from the special session on constrained real-parameter optimization of the 2006 Congress on Evolutionary Computation (CEC) [34]. These well-known benchmark functions are presented in Table 1. In Table 1, ρ is the evaluated ratio between the feasible solution and the search space, *LI* denotes the number of linear inequality constraints, *NI* is the number of nonlinear inequality constraints, *LE* denotes the number of linear equality constraints, *NE* is the number of nonlinear equality constraints, α is the number of active constraints at the optimal solution, and $f\left(x^{*}\right)$ is the objective function value for the optimal solution $x^{*}$.

In order to eliminate random discrepancies, 25 independent runs were performed for each test function. The parameters were set as *NP* = 100, *F* = 0.8, *CR* = 0.9, and *FES* = 350,000. The above parameters were set based on our experiments and they were maintained in all of the runs. In order to make comparisons, three search-bias algorithms, ISR [35], HS [36], and YK [37], and two multi-objective optimization algorithms, ATMES [38] and VY [39], are selected. The results of these seven algorithms are taken directly from [40]. The differences between the optimal values from the six algorithms and the ground truth are listed in Table 2.

It can be noticed that the proposed algorithm has the ability to succeed in finding feasible solutions that are close to the best known solutions for g02, g04, g06, g07, and g08. For g01, g03, and g10, the DE-based algorithm is successful in finding the optimal value. It is indicated that the algorithm can obtain the results that are approximately equal to the optimal solutions for these test functions. 

Only the ATMES, TC, YK, ISR, and HS algorithms are able to find the best known solution for g01. The ISR algorithm achieves the best result for g05. The proposed algorithm has achieved the third best result for g05. g05 has two equality constraints and two inequality constraints, and the optimal value is 5126.4967140071. The rest of the functions have either equality constraints or inequality constraints. This is the difference between g05 and the rest of the functions. According to the free lunch theorem, any algorithm’s elevated performance over one class of problems is exactly offset by its poor performance over another class of problems. However, the proposed algorithm has achieved the best results for the remaining 10 test functions, revealing that the algorithm can consistently find the best solutions in the experiments.

The above observations signify that the mean performance of the proposed algorithm is better than the mean performance of the six algorithms. Therefore, the proposed algorithm is competitive. 

### 4.2. Acquire the Relative Weights among Different Criteria 

The criteria consist of the disaster rate (*C*1), the inundated rate (*C*2), and the complete loss rate (*C*3). The complete loss rate is extremely serious, the inundated rate is very serious, and the disaster rate is serious. According to the AHP theory, the following evaluation matrix *A* can be presented by the experts from the agricultural meteorological disaster field.

$$A=\left|\begin{array}{ccc} 1 & 3 & 7\\ 1/3 & 1 & 5\\ 1/7 & 1/5 & 1 \end{array}\right|$$

Based on the matrix *A*, the min *CIF* is as follows:

$$\min CIF\left(w\right)=\frac{\sum_{i=1}^{3}\left|\sum_{k=1}^{3}\left(A\left(k,i\right)\times w_{k}\right)-3\times w_{i}\right|}{n}$$

**Figure 2:**
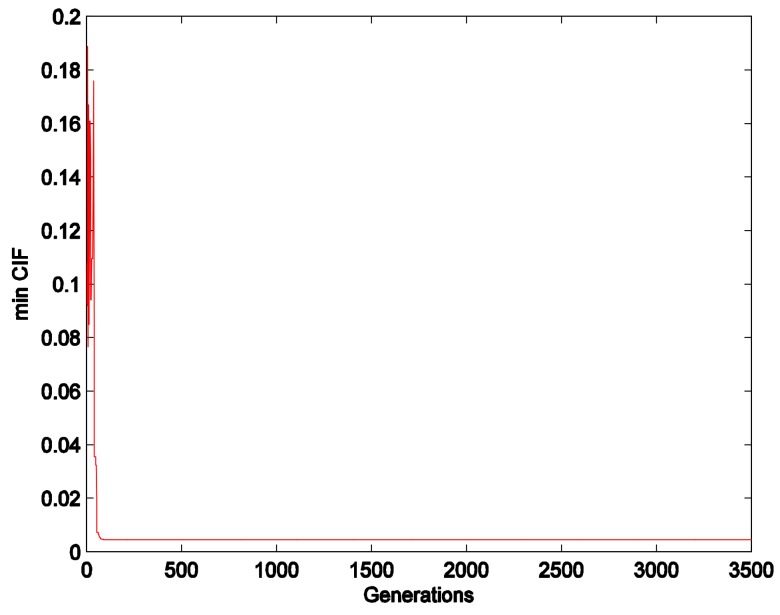
Convergence graph for the min consistent inspection function *(CIF)*.

The *CIF* can be optimized and the weights can be solved for using the proposed algorithm. The convergence graph is presented in Figure 2. The weights are acquired as *W* = [0.6541, 0.2782, 0.0677]. 

### 4.3. Evaluation Results

**Figure 3:**
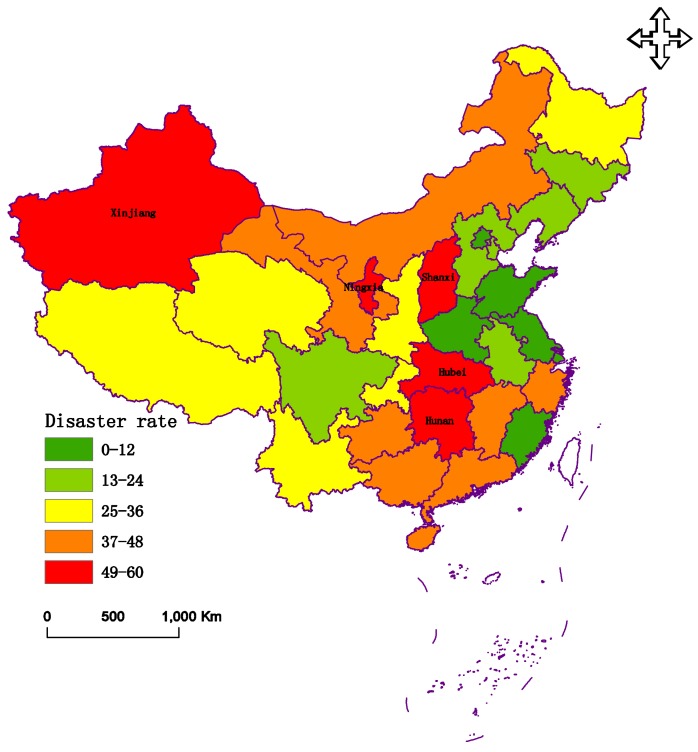
The data of disaster rate (*C*1) in 2008.

**Figure 4:**
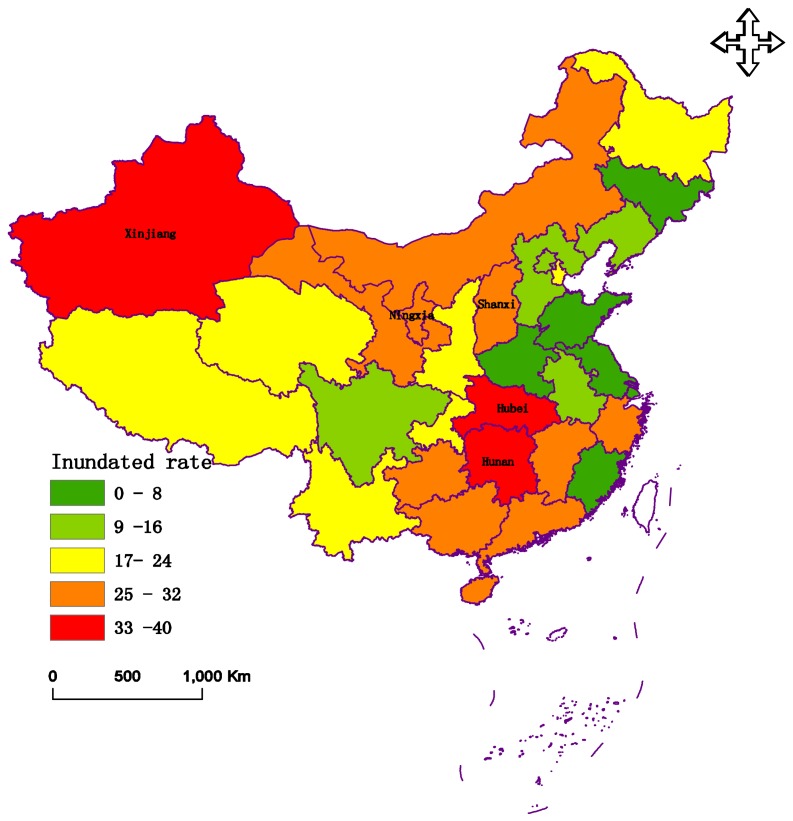
The data of the inundated rate (*C*2) in 2008.

**Figure 5:**
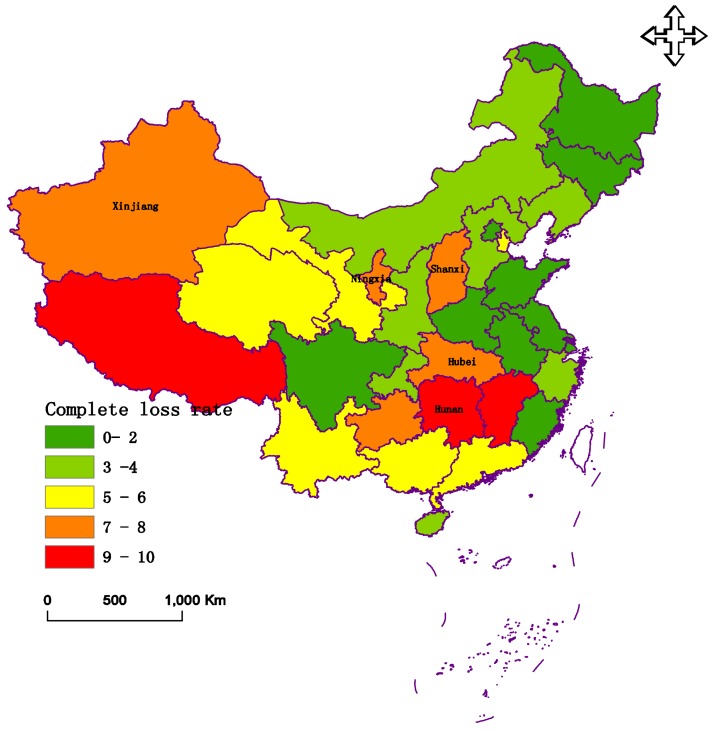
The data of the complete loss rate (*C*3) in 2008.

During the middle and the end of January in 2008, South China experienced a rare and severe cold surge that produced extremely damaging frosts, snow, and ice storms. Prolonged, heavy precipitation occurred over an extensive area of South China [41,42]. There were 31 provinces and cities that were evaluated. The data of these criteria are presented in Figure 3, Figure 4 and Figure 5, respectively. 

Figure 3, Figure 4 and Figure 5 indicate that Hunan Province had the greatest disaster rate and inundated rate, at 59.21% and 37.6%, respectively. The autonomous region of Tibet had the largest complete loss rate. Shanghai experienced the lightest disaster, with rates of 5.4%, 2.1%, and 0%, respectively. 

According to Equation (14), the data can be normalized. The weighted normalized decision matrix can be obtained using Equation (15). Based on Equations (16) and (17), the negative-ideal (*A*^−^) and positive-ideal solutions (*A*^+^) can be found.

**Figure 6:**
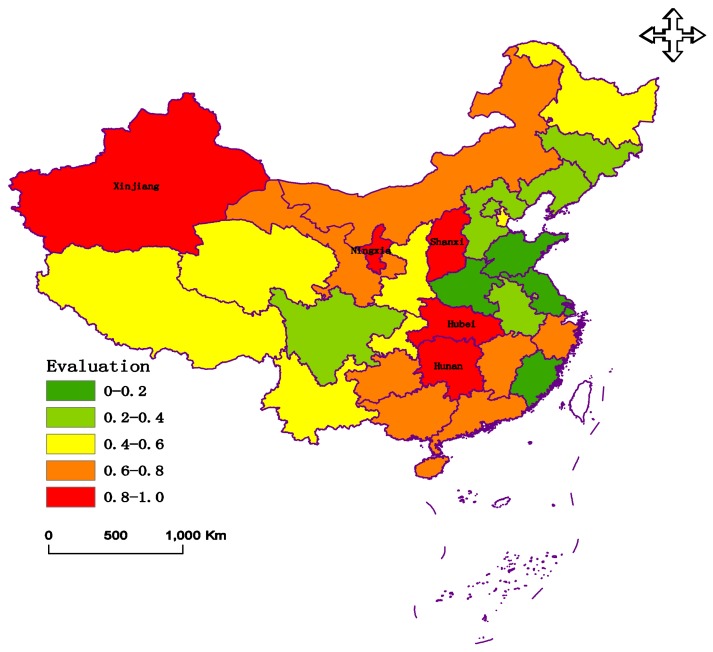
The *CC_j_* values from the evaluation.

*A^−^* = {1.9%, 0.58%, 0%};

*A^+^* = {21%, 10.7%, 3.14%};

Following this, the distances (*D^+^* and *D^−^*) are calculated according to Equations (18) and (19), as demonstrated in Table 3. The disaster degree is determined by the *CC*, which is calculated as shown in Equation (20), and presented in Table 3 and Figure 6.

Table 3 and Figure 6 indicate that the Hunan Province has the largest *CC* value. Therefore, the agricultural disaster in the Hunan Province was the most serious agricultural disaster in 2008.

## 5. Discussion

At the beginning of 2008, a serious ice and snow disaster occurred in parts of South China. This was considered to be a rare nightmare. A massive failure in staple crops occurred in several provinces, with Hunan Province experiencing the most serious failure. The temperature was much lower than that of former years in Hunan Province. The Guangzhou railway was once interrupted.

**Figure 7:**
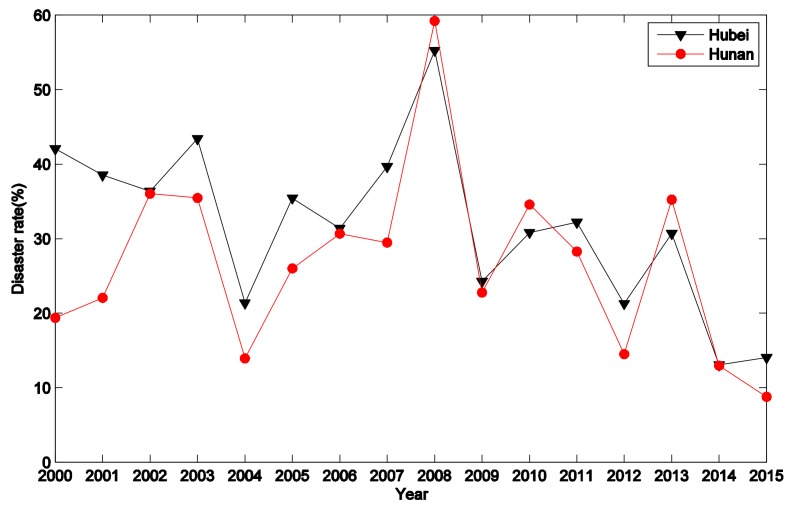
The disaster rate of Hunan Province and Hubei Province from 2000 to 2015.

Hubei Province is located near Hunan Province. The snow disaster also greatly influenced Hubei Province. The evaluation results indicate that it can be ranked as the second most affected province. However, the *CC* values of the other provinces that are located around these two provinces are much lower than the *CC* values of these two provinces. In order to demonstrate the influence of the snow disaster, a time series of the disaster rates from the two provinces is presented in Figure 7. It can be noticed that, except for 2008, the disaster rates of the two provinces are very low. Thus, it can be concluded that the heavy snow had a larger influence on Hunan Province and Hubei Province.

Moreover, the *CC* value of Shanxi Province is 0.8558, indicating that the area suffered from more natural disasters in 2008. The disaster rate of Shanxi Province is more than 50% (up to 58.1%). The inundated rate and the complete loss rate is 27% and 5%, respectively, which is also very high. Shanxi province is restricted by natural conditions. The area lacks rain and water, which is a serious issue. The agricultural environment is very fragile. Floods, droughts, low temperatures and other natural disasters have caused heavy losses every year. 

Meanwhile, the *CC* value of the Ningxia province is 0.8086. The Ningxia Province lies in the east area of Northwest China, which is far away from the ocean. With mild temperatures and semi-dry climate, the climate varies greatly and climate disasters occur frequently. Therefore, it is one of the provinces that is most severely affected by disasters. 

However, the *CC* values of Shanghai, Jiangsu, Fujian, Shandong, and Henan provinces are less than 0.1, indicating that the disasters that occurred around these areas were not severe in 2008. In fact, Henan and Shandong provinces are the two main production areas in China. However, their *CC* values are very small. 

To enhance the defense against agricultural natural disasters, and in an attempt to reduce the losses from agricultural disasters, the relevant departments must firmly establish disaster prevention and anti-disaster measures according to the geographical and climatic features of the region. Moreover, they should make sure that the disaster prevention and alleviation measures are thoroughly implemented.

(1) Speed up the establishment of the disaster warning mechanism, and improve the ability of agricultural natural disaster forecasting.

It is of great importance to make timely, accurate and scientific predictions of agricultural natural disasters using advanced techniques [43]. Before the disaster, a full understanding of the disaster and the necessary preparation is required. During the disaster, strengthening the control of the disaster and resolving the damage are necessary. After the disaster, strengthening the guidance, service, and coordination of agricultural production; disaster relief; and loss compensation are important.

(2) Further strengthen the infrastructure construction of farmland, and enhance the natural disaster prevention ability.

Farmland capital construction is essential for stabilizing grain production and improving the agricultural comprehensive production capacity. Therefore, it is necessary to implement the most stringent farmland protection system. Meanwhile, it is also necessary to increase the intensity of the water conservation facilities, so that the ability of the agricultural natural disaster prevention can be enhanced.

(3) Strongly promote practical agricultural technology, and improve the level of science and technology in order to improve the ability of agriculture to defend against natural disasters.

Further strengthening scientific and technological training; increasing the promotion of comprehensive, practical water saving technology; constantly conducting scientific research; developing new practical technologies; and improving the technological anti-disaster abilities of agricultural stability are required.

(4) Establish the emergency plan for major disasters and improve the ability of emergency responses to natural disasters.

It is necessary to further develop and improve emergency plans for natural disasters. An agricultural production safety and response report system should be established. It will ensure that the information could be timely, accurately, objectively, and comprehensively reported and disseminated.

(5) Increase the support for agricultural disaster recovery and make an effort to reduce agricultural disaster losses.

Farmers could be financially supported by establishing financial funds, special bank credit funds, public welfare investment funds, disaster relief funds, and more. The victims of disasters should be provided with high-quality seeds, seedlings, pesticides, and fertilizers at a low cost, in case the agricultural disaster relief is worsened by fake or shoddy agricultural resources. It is important for victims to fully and accurately grasp the technical essentials and the requirements of breeding varieties.

Moreover, we must speed up the establishment of policy-oriented agricultural insurance institutions and agricultural natural disaster risk protection funds. It is important to appropriately compensate farmers and ensure that they have the capacity to recover production and save themselves after disasters.

## 6. Conclusions

A meteorological disaster is one of the most serious types of natural disasters. It has a serious influence on agriculture. In order to fairly evaluate meteorological disasters, three criteria are designed in order to form the comprehensive indexing system of meteorological disasters. The problem of how to solve for the weights is converted to a single constraint optimization problem. A novel algorithm is proposed to solve for the weights of the criteria. An evaluation model—based on the proposed algorithm and the TOPSIS—is proposed to estimate the agricultural meteorological disaster of 2008 in China. 

In order to validate the performance of the proposed algorithm, 11 testing benchmark functions are selected. The experimental results have indicated that the proposed algorithm is competitive when compared with ATMES, TC, YK, ISR, and HS. The weights of the disaster criteria are obtained by the proposed algorithm. The evaluation results have indicated that the agriculture meteorological disaster of 2008 was serious, especially in Hunan Province, Hubei Province, Shanxi Province, and the Ningxia Autonomous Region. The snow had a great influence on Hunan Province and Hubei province. Suggestions are provided to relieve agriculture meteorological disasters.

## Figures and Tables

**Table 1:** Benchmark test functions.

Test Function	n	Objective Function	ρ	LI	NI	LE	NE	α	$f\left(x^{*}\right)$
g01	13	quadratic	0.0111%	9	0	0	0	6	−15.0000000000
g02	20	nonlinear	99.9971%	0	2	0	0	1	−0.8036191042
g03	10	polynomial	0.0000%	0	0	0	1	1	−1.0005001000
g04	5	quadratic	51.1230%	0	6	0	0	2	−30,665.5386717834
g05	4	cubic	0.0000%	2	0	0	3	3	5126.4967140071
g06	2	cubic	0.0066%	0	2	0	0	2	−6961.8138755802
g07	10	quadratic	0.0003%	3	5	0	0	6	24.3062090681
g08	2	nonlinear	0.8560%	0	2	0	0	0	−0.0958250415
g09	7	polynomial	0.5121%	0	4	0	0	2	680.6300573745
g10	8	linear	0.0010%	3	3	0	0	0	7049.2480205286
g11	2	quadratic	0.0000%	0	0	0	1	1	0.7499000000

**Table 2:** The differences between optimal values from the six algorithms and the ground truth.

Function	Proposed	ATMES	TC	YK	ISR	HS
g01	0 × 10^0^	0 × 10^0^	0 × 10^0^	0 × 10^0^	0 × 10^0^	0 × 10^0^
g02	6.7 × 10^−3^	1.3 × 10^−2^	7.6 × 10^−3^	1.3 × 10^−2^	2.1 × 10^−2^	2.6 × 10^−2^
g03	0 × 10^0^	5.0 × 10^−4^	5.0 × 10^−4^	1.0 × 10^−35^	5.0 × 10^−4^	5.0 × 10^−4^
g04	7.64 × 10^−11^	3.2 × 10^−4^	7.7 × 10^−3^	3.3 × 10^-4^	3.3 × 10^−4^	3.10 × 10^−1^
g05	1.10 × 10^2^	1.15 × 10^0^	1.62 × 10^2^	2.17 × 10^0^	2.86 × 10^−5^	3.47 × 10^2^
g06	3.37 × 10^−11^	1.2 × 10^−4^	1.20 × 10^−4^	6.69 × 10^1^	1.20 × 10^−4^	6.55 × 10^1^
g07	7.26 × 10^−6^	9.8 × 10^−3^	1.68 × 10^0^	1.68 × 10^−2^	2.10 × 10^−4^	1.11 × 10^−1^
g08	8.20 × 10^−11^	9.8 × 10^−3^	1.68 × 10^0^	1.7 × 10^−2^	2.1 × 10^−4^	1.1 × 10^−1^
g09	0 × 10^0^	8.9 × 10^−3^	3.3 × 10^−2^	4.9 × 10^−3^	5.7 × 10^−5^	3.3 × 10^−2^
g10	4.38 × 10^−2^	2.01 × 10^2^	8.43 × 10^2^	1.32 × 10^2^	2.0 × 10^−3^	3.16 × 10^2^
g11	0 × 10^0^	1.0 × 10^−4^	1.0 × 10^−4^	1.0 × 10^−4^	6.1 × 10^−3^	7.71 × 10^−2^

**Table 3:** The *CC_j_* values calculated by Equation (20).

Area	Province and City	*D^+^*	*D^−^*	*CC*
North	Beijing	0.1974	0.0223	0.1015
	Tianjin	0.1647	0.0540	0.2469
	Hebei	0.1834	0.0354	0.1618
	Shanxi	0.0338	0.2006	0.8558
Northeast	Inner Mongolia	0.0993	0.1205	0.5482
	Liaoning	0.1814	0.0369	0.1690
	Jilin	0.1949	0.0237	0.1084
	Heilongjiang	0.1621	0.0566	0.2588
East	Shanghai	0.2183	0	0
	Jiangsu	0.2121	0.0067	0.0306
	Zhejiang	0.0773	0.1450	0.6523
	Anhui	0.1840	0.0344	0.1575
	Fujian	0.2005	0.0184	0.0841
	Jiangxi	0.0694	0.1527	0.6875
	Shandong	0.2153	0.0035	0.0160
South central	Henan	0.2104	0.0089	0.0406
	Hubei	0.0194	0.2029	0.9127
	Hunan	0.0044	0.2177	0.9802
South	Guangdong	0.1003	0.1194	0.5435
	Guangxi	0.0864	0.1346	0.6090
	Hainan	0.0724	0.1513	0.6764
Southwest	Chongqing	0.1567	0.0617	0.2825
	Sichuan	0.1822	0.0365	0.1669
	Guizhou	0.0869	0.1314	0.6019
	Yunnan	0.1423	0.0760	0.3481
	Xizang	0.1437	0.0784	0.3530
Northwest	Shanxi	0.1435	0.0757	0.3553
	Gansu	0.1005	0.1180	0.5400
	Qinghai	0.1438	0.0745	0.3413
	Ningxia	0.0444	0.1876	0.8086
	Xinjiang	0.0467	0.1723	0.7868
